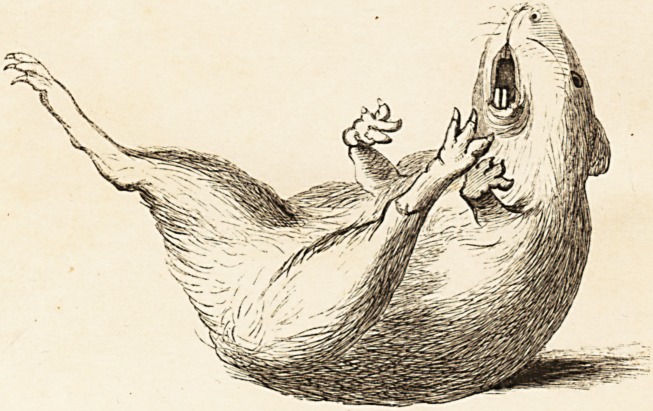# Dr. E. Brown-Séquard on the Physiology and Pathology of the Nervous System

**Published:** 1858-01-01

**Authors:** E. Brown-Séquard


### DR. E. BROWN-SÉQUARD

ON THE

### PHYSIOLOGY AND PATHOLOGY OF THE NERVOUS SYSTEM.

(*With an Illustration of an Epileptic Guinea-pig.*)

The great interest which has been excited among the profession, in London, by Dr. E. Brown-Séquard's Lectures on the Physiology and Pathology of the Nervous System, recently delivered, first at St. Bartholomew's Hospital, and subsequently at the Royal College of Surgeons, has only been commensurate with their importance.

Dr. E. Brown-Séquard has been known for several years as a distinguished and very successful experimental physiologist; but he has not, perhaps, been so well known and appreciated in this country as he would have been, from the fact that his researches have been published in a somewhat disconnected fashion in sundry American, French, and English journals. Hence it has been difficult for many to obtain access to the whole of his writings, and it has never been easy to ascertain the full extent of his researches upon any particular subject.

The lectures which he has recently given in London have been devoted to a summary of his principal researches on the nervous system; and the majority of the experiments by which he seeks to prove the opinions he entertains were exhibited on the living animal during the course of the lectures.

Apart from their subject, Dr. Brown-Séquard's lectures were of singular interest as a psychological study. Although born in a British colony, and now an American citizen, Dr. Brown-Séquard is by maternity half, and by predilection and language wholly, a Frenchman. Speaking English somewhat imperfectly, it might be thought that he would appear at some disadvantage before an English audience; but the vigour of his address and the precision of his descriptions quickly caused the listener to lose sight of any imperfections which might arise from the want of a complete mastery of the English tongue. Indeed, the mind of the lecturer seemed to be almost the more attractively and clearly set forth from the very shortcomings of the guise in which it was shown.

The steady, unfaltering observation,—the careful precautions against error, either from the mind or from the senses,—the checks ever interposed during research, not as stumbling-blocks, but as marks from which to try-back (so to speak), and re-examine, and confirm the truthfulness of the course already run,—the unwearied perseverance, and yet the quiet, soul-warming enthusiasm, which became apparent during the course of the lectures, formed a rare and most instructive study and lesson.

The true character of Dr. Brown-Séquard's researches on the nervous system can only be fully appreciated by a reference to the original memoirs in which they were made known. Many of these memoirs cannot, however, be readily obtained; but the projected publication of the lectures delivered at the Royal College of Surgeons will probably soon place a detailed and connected account of these researches within the reach of most persons.

In this article we propose to give a brief summary of the principal results of Dr. Brown-Séquard's more interesting and important researches on the nervous system.

I. (*a*) It is generally believed by physiologists that the conductors of sensitive impressions in the spinal cord decussate either in the medulla oblongata alone, or in that ganglionic centre, the pons varolii, the corpora quadrigemina, and the crura cerebri,—these structures forming the so-called Isthmus of the Encephalon. Numerous experiments performed by Dr. Brown-Séquard have shown that "*the impressions made on one side of the body are transmitted to the sensorium by the opposite side of the spinal cord*," and that, consequently, the sensitive fibres coming from the trunk and the limbs do not decussate in the medulla oblongata, or the Isthmus of the Encephalon.

The following experiments seem to prove fully the crossed transmission of sensations in the spinal cord. (1) If a lateral half (*i.e.*, the posterior and the antero-lateral columns and the grey matter of one side of the spinal cord) is divided transversely at the level of the tenth costal vertebra, on a mammal, it is soon evident that the sensibility is much diminished in the posterior limb opposite to the side of the section. On the contrary, the sensibility, instead of being lost, appears much increased in the posterior limb on the side where the section has been made. (2) If, instead of one transversal section of the spinal cord, two, three, or many more are made on the same lateral half of that organ, the same results are obtained. (3) If, instead of mere sections, a removal of a part of a lateral half of the spinal cord is effected, the same results are still obtained. (4) If a longitudinal section is made on the part of the spinal cord giving nerves to the posterior extremity, so as to divide that part into two lateral halves, then it is found that sensibility is completely lost in the two posterior limbs, although voluntary movements take place in them. (5) If a similar separation of two lateral halves of the spinal cord is made on the whole part supplying nerves to the anterior limbs, then it is found that sensibility is lost in both these limbs, and that it is only slightly diminished in the posterior limbs. (6) If the same operation is done as in the preceding experiment, and afterwards if a transversal division is made on one of the lateral halves, in the place where it is separated from the other, then it is found that the posterior limb on the side of the transversal section remains sensible, and that the other posterior limb loses its sensibility.

By these experiments the crossing of the sensitive nervous fibres in the cord is very clearly shown. The last three experiments demonstrate directly the crossing; and the transversal sections of a lateral half of the cord prove that sensibility is much diminished in the side of the body opposite to that of the section, consequently they prove also that there is a crossing of a great part of the sensitive fibres.

Numerous cases of injury or disease of a lateral half of the spinal cord in man are on record, in which, while palsy existed on the side of the injury, there was also a greater or less degree of hyperæsthesia on the same side, and anæsthesia existed on the opposite side. These cases, which were previously regarded as being anomalous and inexplicable, are now fully explained by Dr. Brown-Séquard's researches, and they form most weighty evidence in favour of his conclusions.

Further, Dr. Brown-Séquard argues that—

"If the crossing of the sensitive-nerve fibres does not take place in the spinal cord, it must take place somewhere in the medulla oblongata and pons varolii. If it takes place in the medulla oblongata and the pons, what must we find in cases where an alteration exists only in one side of these nervous centres? There must evidently be a diminution of sensibility on both sides of the body, because many fibres belonging to the two sides must necessarily be altered or divided. Let us suppose, for instance, a tumour, as large as a walnut, having altered or destroyed one of the sides of the pons varolii (there are many such cases on record); and let us admit that it is the left side. Now, in this side there are the sensitive fibres of the left side of the body, which have not yet made their decussation, and which make it in the pons, or a little forwards, between the corpora quadrigemina or crura cerebri. These fibres are altered or divided, and, in consequence, the left side of the body must lose a part of its sensibility. But the fibres coming from the right side of the body, and which have made their crossing in the medulla oblongata, and also the fibres from the right side which pass in the pons from the right to the left, must be altered or divided, and, in consequence, the right side of the body must lose a part of its sensibility; so that the two sides of the body, in this hypothesis, must have a diminution of sensibility. A like reasoning might be applied to what must take place when the disease exists in one side of the medulla oblongata, admitting that the decussation of sensitive fibres takes place mostly there; and the same thing might be said also for the parts anterior to the pons varolii, if it were admitted that there takes place the greatest part of the decussation of the sensitive-nerve fibres. Let us now see, then, what are the facts. In almost all the cases where a disease has existed on one side of the medulla oblongata, the pons, &c., there has been a loss or a diminution of sensibility on the opposite side of the body, and no diminution of sensibility on the same side. In cases where there has been an alteration on both sides (in one of the above-named nervous centres), but greater in one than in the other, sensibility was lost in the side of the body opposite to the side of the nervous centre which was most altered, and only diminished on the side of the body opposite to the side less altered in the nervous centre."

The increase of sensibility which occurs when a transversal section of the spinal cord in an animal is made, in the posterior limb on the side of the section, is a very interesting phenomenon. Its early development is due, in part, to absorption of oxygen from the atmosphere by the exposed spinal cord; but the cause of the hyperæsthesia, as dependent upon the injury done to the spinal cord, is, according to Dr. Brown-Séquard, paralysis of the vascular nerves. The palsied blood-vessels dilate, more blood is admitted to them, nutrition becomes more active, and, as a consequence, the vital properties, both of nerves and muscles, are increased. The effects, indeed, upon the circulation and nutrition of the parts below and upon the same side as a transversal section of the cord, are similar in character to those observed after section of the sympathetic nerve in the neck. It is worthy of remark, that the hyperæsthetic condition of the paralysed parts continues so long as the animal lives, and the functions of the injured cord are not restored.*

(*b*) The posterior columns of the spinal cord do not perform that important part in the transmission of sensitive impressions to the encephalon which some physiologists teach. Sensation is not destroyed by division of the posterior columns, and their integrity does not prevent loss of sensation. On the other hand, so long as a small portion of the central grey matter of the cord remains intact, sensitive impressions are transmitted to the encephalon; but complete division of the central grey matter deprives all parts below the section of all (save a very obscure) sensibility. Again, any injury of the antero-lateral columns does not affect the transmission of sensitive impressions in the cord. Hence it follows (as was partly surmised by Dr. Todd) that the central grey matter of the cord is the principal conductor of sensitive impressions from the trunk and limbs to the encephalon.

After, however, complete division of the central grey matter of the cord, certain sensitive impressions are still transmitted, although very obscurely, to the brain from the parts below the section; and Dr. Brown-Séquard believes that the anterior columns contribute positively, though but very little, to the transmission of sensitive impressions. This property seems to be possessed by a thin layer forming the sur-

* "Experimental Researches applied to Physiology and Pathology." By E. Brown-Séquard. New York. 1853. c. xx. This work consists of a series of papers reprinted from the "Philadelphia Medical Examiner."—"Experimental and Clinical Researches on the Physiology and Pathology of the Spinal Cord, and some other parts of the Nervous Centres." 8vo. Richmond (U.S.). 1855.—"On the Spinal Cord as a Leader for Sensibility and Voluntary Movements."—Proceedings of the Royal Society," vol. viii. p. 591.

face of these columns in contact with the grey matter. The fibres of this layer are totally deprived of sensibility.*

According to Dr. Brown-Séquard, sensitive impressions are transmitted to the encephalon in the following manner:—

"At their arrival in the spinal marrow, the sensitive impressions pass by the posterior columns, the posterior grey horns, and probably by the lateral cords. In these different parts of the spinal cord the sensitive impressions mount or descend (it may be shown that they will pass either upwards or downwards in the posterior columns), and after a short tract towards the encephalon or in the opposite direction, they quit these parts to enter the grey central substance, in which, or by which, they are finally transmitted to the encephalon."†

(*c*) When the spinal cord is cut slowly across from behind forwards, a gradual augmentation of sensibility is observed in all the parts below the incision until a certain limit is attained (the centre of the cord), beyond which limit, as the incision is advanced, sensibility diminishes gradually until it is extinguished. The changes in degree of sensibility occur simultaneously in all the parts below the incision.

When the spinal cord is divided gradually from side to side, the changes in degree of sensibility which follow in the parts below the division also occur simultaneously, and in an equal degree, in all the parts affected.

From these experiments it follows that the conductive elements of sensitive impressions are not disposed in columns or layers, each of which is connected with a definite portion of the skin, the muscles, &c. (as has been supposed by some physiologists); for if this were the arrangement, on a gradual section of the cord, a notable diminution of sensibility would be observed in some parts of the skin, or in some groups of muscles, &c., below the section, other parts of the skin and other groups of muscles being unaffected.

It results from Dr. Brown-Séquard's experiments, that every portion of the recipient and conductive elements of any segment of the spinal cord, takes a part in the reception and conduction of sensitive impressions from every portion of the body with which it is connected. For example, the different conductive elements proceeding from the surface of the anterior and from the surface of the posterior parts of the body, as well as the conductive elements of the intermediate parts, are distributed in all the conductive parts of the spinal cord, before and behind. The conductive elements proceeding, also, from the external surface of the thigh or arm, and from the internal surface of these members, as

* The property of being sensitive and that of conveying sensitive impressions are distinct one from the other; nerve-fibres employed to convey sensitive impressions may be deprived of sensibility.

† "Comptes Rendus des Séances et Mémoires de la Société de Biologie," t. xii. 1854.—"Proceedings of the Royal Society," vol. viii. p. 591.

well as from the intermediate parts, are distributed in all the conductive portions of the spinal marrow, transversely, in the lateral half of the opposite site. On the other hand, if a very limited zone of skin be imagined—a zone represented, it may be supposed, by a thousand conductive elements in the spinal marrow—these one thousand elements are disseminated in a lateral half of this nervous centre in such a fashion that they are present everywhere—before, behind, in the middle, near or far from the borders of the zone by which the transmission of sensitive impressions is effected in this organ.

In short, *the smallest portion of the conductive zone, in a lateral half of the spinal cord, contains the conductive elements of sensitive impressions proceeding from every point of the body on the opposite side, placed below this little portion of the cord.* Again, *the impressions proceeding from every portion of a lateral half of the body are transmitted to the encephalon by conductive elements distributed in every portion of the conductive zone of the lateral half of the spinal marrow on the opposite side.*

These data explain how it happens that sensibility is so rarely lost in cases of softening or of other alterations of the spinal cord, because so long as a healthy portion of the conductive elements of the cord remains, sensitive impressions will be transmitted from the parts of the body which are situated below the lesion in the cord. These data also explain why, in cases of hyperæsthesia or of anæsthesia of different degrees, dependent upon lesions of the spinal cord, the increase or diminution of sensibility is found almost equally distributed everywhere in the parts which are situated below the seat of the lesion.

Anæsthesia or hyperæsthesia which is limited to a small portion of the extremities, or trunk, is dependent upon a lesion of the encephalon, or of a nerve, and not of the spinal cord.*

(*d*) In mammals, birds, and other animals, after a transverse section of a lateral half of the spinal cord, voluntary motion is not entirely lost in the parts situated on the same side below the sections. In these animals there can be little doubt that a few motor fibres decussate in the spinal cord. There is no evidence of the existence of such a decussation in man. In him the voluntary motor fibres decussate in the medulla oblongata. If the decussation took place in the pons varolii, as stated by Valentin, Longet, and others, disease of the pons would cause symptoms different from those ordinarily observed in affections of that portion of the nervous centres. For example, if one-half of the pons varolii were diseased, and the decussation of voluntary motor fibres

* "Nouvelle Recherches sur la Physiologie de la Moëlle Épinière," par Dr. E. Brown-Séquard.—"Journal de la Physiologie de l'Homme et des Animaux," publié sous la direction du Dr. E. Brown-Sequard, No. 1, p. 139.

took place in that organ, both sides of the body would be partially paralysed. But such is not the case, for the paralysis which is occasioned by disease of one-half of the pons varolii is confined to one side of the body.

Dr. Brown-Séquard considers that the voluntary motor nerve-fibres are distributed in the spinal cord in the following manner:—

The anterior pyramids of the medulla oblongata contain most of the voluntary motor nerve-fibres. In the cervical region of the spinal cord, the voluntary motor nerve-fibres are mostly in the lateral columns and the anterior grey cornua. In the dorsal and lumbar regions of the spinal cord, these nerve-fibres are in the anterior columns and in the grey matter.

There can be no doubt that the whole of the voluntary motor nerve-fibres decussate in the medulla oblongata; but the whole of the fibres of the anterior columns of the cord do not decussate there, some passing on without decussation towards the encephalon. These fibres, Dr. Brown-Séquard regards as being motor, but not voluntary motor, and he believes that fibres of this character exist plentifully in the encephalon. He derives his principal reason for this conclusion from the pathological fact that convulsion may be caused on one side of the body by the same encephalic lesion which has caused paralysis on the opposite side.*

(*e*) Dr. Brown-Séquard's views on the crossed transmission of sensitive impressions in the spinal cord throw great additional light upon paralytic affections. If it be assumed that the sensitive nerve-fibres of the trunk and limbs decussate in great part, if not wholly, in the spinal cord, and not in the isthmus of the encephalon; and if it be also assumed that the voluntary motor nerve-fibres decussate in the medulla oblongata,—then it would follow that, according to the seat of an alteration in the cerebro-spinal axis producing a paralysis, three different kinds of paralysis may exist:—

"(1) The alteration being in any part of the encephalon except the inferior portion of the medulla oblongata, the paralysis of voluntary motion and sensibility will exist on the side of the body opposite to the side of the disease.

"(2) The alteration occupying an entire lateral half of the inferior portion of the medulla oblongata at the level of the decussation of the pyramids, the paralysis of voluntary movement exists on both sides of the body, but incomplete; and the paralysis of sensibility exists only on one side, and it is that opposite to the side of the disease.

"(3) The alteration occupying the entire thickness of a portion of a lateral half of the spinal cord, the parts of the body situated behind it, at the same side, are paralysed of voluntary movement, and the corresponding parts on the other side are paralysed of sensibility."†

* "Experimental and Clinical Researches on the Physiology and Pathology of the Spinal Cord."—"Proceedings of the Royal Society," vol. viii. p. 591.

† "Experimental and Clinical Researches on the Physiology and Pathology of the Spinal Cord."

(*f*) Paralysis of sensibility occasionally exists in different parts of both sides of the body, produced by an alteration in only one side of the spinal cord. Three different kinds of paralysis may be described as produced by an alteration in a lateral half of the cord, and all characterized by the existence of paralysis of movement on one side of the body, and a more or less extended paralysis of sensibility on the two sides of the body. (1) If an alteration able to produce paralysis exists in the whole thickness of a lateral half of the cord, in the entire extent of the part from which come all the nerves going to one of the upper limbs, there will be paralysis both of movement and sensibility in that limb, and paralysis of movement in the trunk and the inferior limbs on the same side of the body, and, besides, paralysis of sensibility in the opposite side of the body (limbs and trunk). (2) If an alteration able to produce paralysis exists in the whole thickness and length of a lateral half of the cord which gives off all the nerves going to one of the inferior limbs, there will be paralysis both of movement and of sensibility in that limb, and only paralysis of sensibility in the opposite limb. (3) If an alteration able to produce paralysis exists in the whole thickness and in the whole length of a lateral half of the cord, the symptoms will be a paralysis of movement in the side of the body corresponding to the side altered in the cord, and a paralysis of sensibility on the two sides of the body (neck, trunk, and the four limbs).*

(*g*) Local paralysis of sensibility consequent upon an injury of the back, and on the side of the injury, is probably due to undue stretching of the roots of the posterior spinal nerves.

II. The medulla oblongata is usually regarded as the nervous centre of the respiratory movements, and as being, of all portions of the nervous system, that which is most essential to life. It would seem probable, therefore, that the removal of such an organ, even in cold-blooded animals, would be followed by speedy death. Such is not, however, the result of the operation; and Dr. Brown-Séquard ascertained that batrachia would live, under favourable conditions, more than four months after the loss of the medulla oblongata. During all that time the animals operated on remained seemingly in good health.

The duration of life after ablation of the medulla oblongata differs in different species of animals; and it may be reckoned by *months* for batrachia, by *weeks* for some reptilia, by *days* for other reptilia and for fishes, by *hours* for hybernating mammals, and by *minutes* (three to forty-six) for birds and non-hybernating mammals.

* "Experimental and Clinical Researches on the Physiology and Pathology of the Spinal Cord."

In animals deprived of the medulla oblongata, death is principally caused by insufficiency of respiration.*

Flourens, from the results of certain experiments which he made, arrived at the conclusion that the little V-shaped collection of grey matter which is situated at the neb of the calamus, in the fourth ventricle, is the prime centre of the respiratory mechanism; and also, that this seemingly insignificant spot is essential to the integrity of the nervous system, and of life itself. Hence he called this little collection of grey matter the "*nœud vital.*" Dr. Brown-Séquard has made a series of experiments with direct reference to Flourens' opinions on the "*nœud vital,*" and the following interesting results have been arrived at:—

(1) Death is not always an immediate result of ablation of the "*nœud vital.*" (2) When death occurs suddenly after this ablation, it is due in part to the sudden stoppage of the movements of the heart. (3) Irritation of the parts in the vicinity of the "*nœud vital,*" as well as ablation of that point, produces sometimes arrest or enfeeblement of the movements of the heart. (4) After section of the pneumogastric nerves, ablation of the "*nœud vital*" occasions a sudden stoppage of the movements of the heart. (5) It is not in consequence of the absence of the "*nœud vital*" that the respiratory movements become sometimes arrested after ablation of that little organ, but rather in consequence of an irritation of the medulla oblongata of the same kind as follows galvanization of the pneumogastric nerves. (6) Irritation of the parts adjacent to the "*nœud vital*" sometimes causes stoppage of the respiration, when the "*nœud vital*" is not injured. (7) Respiration and circulation may continue with vigour and regularity during a great number of days, after ablation of the "*nœud vital;*" whence it results that the point is neither the centre of origin of a pretended vital force, nor the prime motive centre of the respiratory mechanism. (8) Voluntary movements and the functions of the senses persist often after ablation of the "*nœud vital.*" (9) The "*nœud vital*" does not appear to be essential to life.

Dr. Brown-Séquard has concluded, from his experiments upon the medulla oblongata, that the nervous centre of the respiratory movements is not limited to the parts in which modern physiologists have almost unanimously fixed it.†

III. Dr. Cl. Bernard discovered the curious fact that, after section of the sympathetic nerve in the neck, the face and the ear—particularly the latter—on the same side as the section, became warmer and more

* "Experimental Researches applied to Physiology and Pathology," c. xvi.

† "Causes de Mort après l'Ablation du Nœud Vital."—Brown-Séquard's "Journal de la Physiologie," No. 2.

sensitive than on the other side. Dr. Brown-Séquard repeated Dr. Cl. Bernard's experiments, and added several particulars to those which he had discovered. Moreover, Dr. Brown-Séquard ascertained that, after section of a lateral half of the spinal cord in the dorsal region, phenomena are observed in the posterior limb on the corresponding side, similar in character to those observed on the side of the face after section of the cervical sympathetic.

The following is a summary of the results of these important experiments:—

| *Section of the Cervical Sympathetic: its effects on the corresponding side of the face.* (Cl. Bernard and Brown-Séquard.) | *Section of a lateral half of the Spinal Cord in the Dorsal Region: its effects on the posterior limb on the corresponding side.* (Brown-Séquard.) |
|---|---|
| 1. Blood-vessels dilated (paralysed). | 1. The same effect. |
| 2. As a consequence, more blood. | 2. The same effect. |
| 3. Elevation of temperature. | 3. The same effect. |
| 4. Sensibility slightly increased. | 4. Very much increased. |
| 5. Ditto, lasting longer there than on the other side, when the animal is chloroformized. | 5. Lasting longer than anywhere else during chloroformization. |
| 6. Sensibility lasting longer there than on the other side during agony. | 6. Longer than anywhere else during agony. |
| 7. Many muscles contracted. | 7. A state of slight contraction of the muscles. |
| 8. Absorption more rapid. | 8. The same effect. |
| 9. Increase of sweat and other secretions. | 9. Increase of sweat. |
| 10. Reflex movements last longer than elsewhere after death. | 10. The same effect. |
| 11. After poisoning by strychnia the first convulsions take place. | 11. The same effect. |
| 12. A galvanic current too weak to excite convulsions elsewhere may act there. | 12. The same effect. |
| 13. The motor nerves, after death, remain longer excitable there than on the other side. | 13. The motor nerves, after death, remain *notably longer* excitable there. |
| 14. The muscles, after death, remain longer contractile there than on the other side. | 14. The muscles, after death, remain much longer contractile there. |
| 15. The contractility of blood-vessels is greater, and lasts longer. | 15. The same effect. |
| 16. The galvanic muscular current (as ascertained with the rheoscopic frog) is stronger, and lasts longer than on the other side. | 16. The same effect (more marked). |
| 17. Cadaveric rigidity appears later there than on the other side, and it lasts longer. | 17. Cadaveric rigidity appears *notably later* there than elsewhere, and lasts longer. |
| 18. It is easier to regenerate there than on the other side the vital properties of nerves and muscles, by in- | 18. The same effect (more marked). |

| | |
|---|---|
| jections of red blood a short time after they have disappeared. | |
| 19. Putrefaction comes on later, and seems to progress more slowly there than on the other side. | 19. The same effect (more marked). |

The whole of these remarkable phenomena, according to Dr. Brown-Séquard, arise from paralysis of the vascular nerves, and consequent dilatation of the blood-vessels. An increased quantity of blood finds its way to, and is contained in, the dilated vessels, the temperature becomes greater, nutrition is more active, and, as a consequence, the vital properties of nerves, muscles, and blood-vessels are increased.

The side of the face opposite to that on which the sympathetic in the neck has been divided, and the posterior limb of the side on which the spinal cord is uninjured, in the experiments of which the foregoing are the results, received less blood than usual, the temperature was lessened, nutrition was less active, and the vital properties of both nerves and muscles were diminished.*

IV. The rotatory movements which occur after injuries to certain portions of the nervous centres have long interested physiologists. Turning or rolling, to the one side or the other, may be occasioned by an injury to any portion of the cerebro-spinal centres, except the cerebral hemispheres, the cerebellum, the corpora striata, the corpus callosum, the spinal marrow, and the olfactive and optic nerves. Rolling is ordinarily produced by injury of some parts, and turning by injury of others; but both kinds of movement may occur after injury of one part of the encephalon only. Dr. Brown-Séquard believes that the principal cause of these rotatory movements is the existence of a convulsive contraction in some of the muscles on one side of the body. This convulsive contraction is found in every case of circulatory and rotatory movement, and it is dependent upon the irritation produced in the injured portion of the encephalon.†

V. Of all Dr. Brown-Séquard's discoveries none has excited so much interest, none is more suggestive than that in which he has shown that certain injuries of the spinal cord, in different species of animals, are followed, in a few weeks' time, by "epilepsy, or at least a disease resembling epilepsy." This result is occasioned by the following kinds of injury to the spinal cord: 1st. A complete transversal section of a lateral half of this organ. 2nd. A transversal section of its two posterior columns, of its posterior cornua of grey matter, and of a part of the lateral columns. 3rd. A transversal section of either the posterior columns or the lateral, or the anterior alone. 4th. A complete

* "Proceedings of the Royal Society," vol. viii. p. 594.

† "Experimental Researches applied to Physiology and Pathology," c. v.

transversal section of the whole organ. 5th. A simple puncture. Of all these injuries, the first, the second and the fourth seem to have more power to produce epilepsy than the others. The first particularly, *i.e.*, the section of a lateral half of the spinal cord, seems to produce constantly this disease in animals that live longer than three or four weeks after the operation. After a section of either the lateral, the anterior, or the posterior columns alone, epilepsy rarely appears; and it seems that in the cases where it has been produced, there has been a deeper incision than usual, and that part of the grey matter has been attained. In other experiments, few in number, section of the central grey matter (the white being hardly injured) has been followed by this convulsive disease. It has occasionally, but very rarely, occurred after a single puncture of the cord. It is particularly after injuries to the part of the spinal cord which extends from the seventh or eighth dorsal vertebra to the third lumbar, that epilepsy appears.

The affection usually begins during the third or fourth week after the injury. At first the fit consists only in a spasm of the muscles of the face and neck, either on one or the two sides, according to the transversal extent of the injury. After a few days the fit becomes more complete, and the convulsions extend to every portion of the body which is not paralysed. The parts convulsed vary greatly according to the seat of the injury.

The " convulsions may come on spontaneously or after certain excitations. The most interesting fact concerning these fits is that it is possible, and even very easy, to produce them by two modes of irritation. If we take two guinea-pigs, one not having been submitted to any injury of the spinal cord, and the other having had this organ injured, we find, in preventing them from breathing for two minutes, convulsions come on in both; but if they are allowed to breathe again, the first one recovers almost at once, while the second continues to have violent convulsions for two or three minutes, and sometimes more. There is another mode of giving fits to the animals which have had an injury to the spinal cord. Pinching of the skin in certain parts of the face and neck is always followed by a fit. If the injury to the spinal cord consists only in a transversal section of a lateral half, the side of the face and neck which, when irritated, may produce the fit, is on the side of the injury; *i.e.*, if the lesion is on the right side of the cord, it is the right side of the face and neck which are able to cause convulsions, and *vice versâ*. If the two sides of the cord have been injured, the two sides of the face and neck have the faculty of producing fits, when they are irritated. In other portions of the body but a portion of the face and neck has this faculty." The portion of the face and neck having this power is contained within a zone limited by the four following lines: one uniting the ear to the eye; a second from the eye to the middle of the length of the inferior maxillary bone; a third, which unites the inferior extremity of the second line to the angle of the inferior jaw; and a fourth, which forms half a circle and goes from this angle to the ear, and the convexity of which approaches the shoulder. The property which the portion of skin included within the lines here described, possesses of occasioning fits on being irritated cannot be ascribed to excessive sensibility, because, when the injury exists only in one of the lateral halves of the cord, the face and neck on the other side have not the power of producing fits, whatever is the degree of irritation upon them; in the same case, the posterior limbs on the side where the cord is injured, is in a state of hyperæsthesia, and nevertheless, the most violent irritations upon this limb do not produce fits; and it is sometimes sufficient to touch the face or the neck, or even to blow upon them, to produce the fits.

Dr. Brown-Séquard thinks that these fits ought to be considered as epileptic.

"The following description," he writes, "of these convulsions will show that, if they are not positively epileptic, they are at least epileptiform. When the attack begins, the head is drawn first, and sometimes violently towards the shoulder by the contraction of the muscles of the neck, on the side of the irritation; the mouth is drawn open by the contraction of the muscles of the neck which are inserted upon the lower jaw; and the muscles of the face and eye (particularly the orbicularis) contract violently. All these contractions usually occur simultaneously. Frequently at the same time, or very nearly so, the animal suddenly cries with a peculiar hoarse voice, as if the passage of air was not free through the vocal chords, spasmodically contracted. Then the animal falls, sometimes on the irritated side, sometimes on the other, and then all the muscles of the trunk and limbs that are not paralysed become the seat of convulsions, alternately clonic and tonic. The head is alternately drawn upon one or the other side. All the muscles of the neck, eyes, and tongue contract alternately. In the limbs, when the convulsions are clonic, there are alternative contractions in the flexor and extensor muscles. Respiration takes place irregularly, on account of the convulsions of the respiratory muscles. Almost always there is an expulsion of fæcal matters, and often of urine. Sometimes there is an erection of the penis, and even ejaculation of semen."

These fits differ in certain particulars from epilepsy in man. The animals occasionally appear to retain their sensibility during the fits; no foam is found at the mouth; and the fits most commonly consist of a series of convulsive attacks, in the intervals of which the animals can rise and stand upon their feet. But these differences, Dr. Brown-Séquard thinks, ought not to prevent our considering the fits as true epileptic fits. "Not only the convulsions resemble those of true epilepsy, but the fits are not mere accidents; and they come by series of two or three, once a-week, once a-day, or even ten or twenty times a-day, and the disease lasts for years. Besides, we find, after long and violent fits, that these animals are, for a time, in a state of drowsiness, like men after epileptic convulsions. It seems rational to conclude, from this discussion, that if the convulsions of these animals are not truly epileptic, they are at least epileptiform."

We have recently had frequent opportunities of witnessing these epileptiform seizures in a guinea-pig, a lateral half of the spinal cord of which, in the lower part of the dorsal region, had been completely divided, transversely, by Dr. Brown-Séquard, about four months ago. The wound in the vertebral column is healed up, fresh osseous matter having been deposited, and voluntary motion has been partially regained in the leg which has been paralysed by the section of the cord. The paralysed leg is highly hyperæsthetic, while in the opposite limb complete anæsthesia exists (an interesting illustration of the crossing of the sensitive nerve-fibres in the spinal cord). Slight irritation upon the skin of the face and neck within the small space* described by Dr. Brown-Séquard, immediately excites a convulsive attack. No better description of the fit can be given than that which has been given by Dr. Brown-Séquard, and which we have already quoted. The great interest of the phenomena connected with these convulsive attacks, has led us to conceive that a sketch of this pig, in a state of repose, and also a sketch of the animal in one of the most ordinary phases of a convulsive seizure, might prove interesting to those of our readers who have not had an opportunity of witnessing Dr. Brown-Séquard's experiments. In the accompanying plate (*see Frontispiece*) the first figure represents the guinea-pig in a quiescent state, the paralysed right-hand leg being protruded in a half-helpless fashion behind the animal: in the second figure the animal is portrayed in a moment of violent convulsion, which had been excited by pinching the skin above the angle of the right inferior jaw.

From his experiments on animals, and from pathological facts observed in man, Dr. Brown-Séquard has concluded that—(1) There cannot be any doubt that in animals certain injuries to the spinal cord frequently produce an epileptiform affection, if not true epilepsy. (2) That in man there are a great many cases which seem to prove that alterations of the spinal cord may cause epilepsy.

The *aura epileptica* in man would seem to be somewhat analogous to the sensory condition originating in the skin and face of animals made epileptic by injury of the spinal cord. "In them, as well as in

* This portion of the skin in all epileptic guinea-pigs is much infested with lice, some modification either of the surface or its secretion seemingly attracting these parasites to this point.

man (when there is a real aura), the trunks of the nerves seem not to possess the faculty of producing fits, whereas their ramifications in the skin, or in the muscles, have this power. In (epileptic) animals as well as in man, if there is an interruption of nervous transmission between the skin and the nervous centres, fits are no more seen, or at least their number is very much diminished. Many cases of epilepsy with an evident aura epileptica, are on record, in which there has been either a diminution of the fits, or more frequently a complete cure, after the interruption of nervous transmission between the starting point of the aura and the nervous centres. In these cases, the following various means have been employed with complete or partial success, either against the aura epileptica or against its production: 1st, ligature of a limb or a finger; 2nd, sections of one or more nerves, and amputation of a limb, or of other parts of the body; 3rd, elongation of muscles which are the seat of the aura; 4th, cauterization, by various means, of the part of the skin from which the aura originates."

From what occurs in animals after an injury to the spinal cord, and from some cases observed in man, it is not improbable that the existence of a particular spot capable of producing fits, when irritated, is not uncommon in epileptic patients. The existence of this spot may not be known to the patient, and it may be found only after a careful search.

In epileptic animals the brain is not essential to epileptiform convulsions. After it has been taken away the fit may be produced almost as easily as before the operation, by pinching the skin of the face and neck. Epilepsy in these animals has its seat in either the pons varolii, the medulla oblongata, or the spinal cord, or in these three parts together.

Dr. Brown-Séquard believes that epilepsy depends in great measure on an increased reflex excitability of certain parts of the cerebro-spinal axis. Many of his experiments, he thinks, " have shown that the reflex faculty of the cerebro-spinal axis is composed, as the muscular contractility is, of two elementary vital properties, one of which he calls the *reflex excitability*, and the other the *reflex force*. The cerebro-spinal axis may have a great reflex force, and very little excitability. It may, on the contrary, have an excessive reflex excitability with very little reflex force. In almost all epileptics, if not in all, the reflex excitability is increased, while the reflex force is rarely above, and often below, its normal degree."

The sudden cessation of the functions of the brain in epilepsy, may be explained by supposing that the branches of the sympathetic going to the blood-vessels of the brain proper, suffer from some irritation. As a consequence of this irritation, contraction occurs in these bloodvessels, particularly in the small arteries. This contraction expelling the blood, the brain proper loses at once its functions, just as it does in complete syncope. The continuation of insensibility after the cessation of convulsions is due to, and in proportion to, the deficient oxygenation of the blood supplied to the brain proper.

From a careful consideration of the phenomena observed in epileptic animals and in the epilepsy of man, Dr. Brown-Séquard lays down the following propositions (among others) for the treatment of the disease: (1) The first thing to be done in a case of epilepsy is to find out if its origin is peripheric. The state of all the organs must be inquired into as completely as possible. (2) If it be ascertained that epilepsy is of peripheric origin, proper means must be employed to separate the nervous centres from that origin, or to remove the cause of the excitation entirely. Leaving aside what relates to the viscera, the application of ligatures ought to be tried first. (3) If ligatures fail, this is no reason for despairing of other means having the same object. The nerve animating either the part of the skin from which the aura originates, or the muscle or muscles which are first convulsed, must be laid bare, and sulphuric ether thrown upon it. This might, perhaps, be sufficient to cure the affection; if it is not, then the nerve must be divided. (4) Sometimes blisters, setons, caustics, &c., in the neighbourhood of a part which is the origin of an aura, may be sufficient to cure, but these means have not the same efficacy as the application of a red-hot iron. (5) The best means of treating epilepsy seem to consist in the application of a series of moxas along the spine, and particularly the nape of the neck. (6) The nutrition of the nervous centres may be modified, and thereby epilepsy cured, principally by the medicines which act on the blood-vessels, such as strychnia, but particularly by those which determine contractions in these vessels, such as atropia, ergot of rye, &c. (7) Hygienic means are as important as the treatment, and sleeplessness ought to be as much combatted as the disease itself.*

In the preceding article we have endeavoured to give in a small compass a correct notion of the character and results of Dr. Brown-Séquard's more interesting and important researches on the Nervous System; and in order to do this more completely, we have avoided any critical examination of his conclusions and opinions.

* "Researches on Epilepsy." (Republished from the "Boston Medical and Surgical Journal.") Boston (U.S.). 1857.

## Figures and Tables

**Figure f1:**
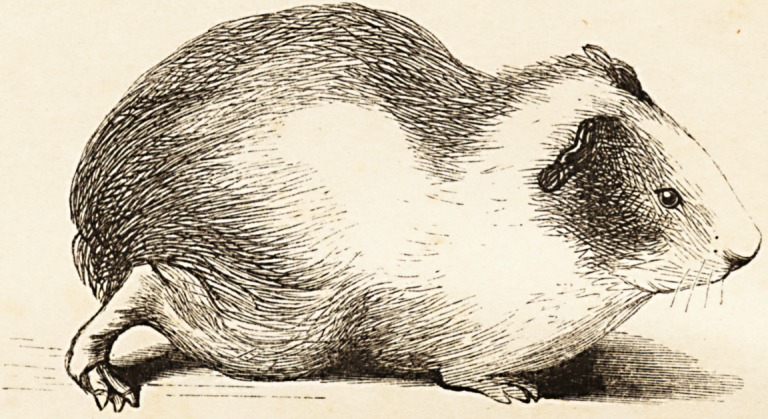


**Figure f2:**